# Volume Conduction Influences Scalp-Based Connectivity Estimates

**DOI:** 10.3389/fncom.2016.00121

**Published:** 2016-11-22

**Authors:** Clemens Brunner, Martin Billinger, Martin Seeber, Timothy R. Mullen, Scott Makeig

**Affiliations:** ^1^Institute of Psychology, University of GrazGraz, Austria; ^2^BioTechMed-GrazGraz, Austria; ^3^Department of Otolaryngology, Hannover Medical SchoolHannover, Germany; ^4^Institute of Neural Engineering, Graz University of TechnologyGraz, Austria; ^5^Qusp LabsSan Diego, CA, USA; ^6^Swartz Center for Computational Neuroscience, University of California, San DiegoSan Diego, CA, USA

**Keywords:** Electroencephalographic, connectivity, inverse problem, directed transfer function, volume conduction, source analysis

## Introduction

Electroencephalographic (EEG) signals recorded from the scalp surface are generally highly correlated. Each channel is a linear mixture of concurrently active brain and non-brain electrical sources whose activities are volume conducted to the scalp electrodes with broadly overlapping patterns (Nunez et al., 1997). This property is particularly relevant to connectivity analyses, which seek to detect and characterize active interactions between brain regions. Therefore, meaningful connectivity patterns can be derived only from measures of cortical source activities and not directly from EEG channel activities (Michel and Murray, 2012). However, this poses a serious problem, since estimating the nature, number, brain (or non-brain) locations, and time courses of the active sources contributing to the scalp EEG is not straightforward (Baillet et al., 2001).

Several methods have been proposed to estimate source activities from multi-channel EEG recordings, thereby removing the confounding effects of volume conduction. These methods can be grouped into three categories: (a) simple spatial filters that seek to reduce correlations between scalp channels based on idealized assumptions; (b) more complex spatial filters that seek to estimate net activities within ROIs based on detailed neurophysiological head models; and (c) blind spatial source separation methods that seek to separately identify source signals by exploiting source signal information differences. Whereas simple spatial filters such as bipolar derivations and Laplacian filters can reduce, to some extent, correlations among scalp-recorded channels (Fisch, 2012), more sophisticated spatial filtering methods use inverse imaging methods to estimate the time courses of cortical sources in given or estimated region of interest (ROI) (Baillet et al., 2001). Blind source separation techniques, in particular independent component analysis, by contrast, learn spatial filters from the EEG time courses that separate the data into constituent independent source activities. Their corresponding brain (or non-brain) locations can then be estimated using neurophysiological inverse imaging methods (Makeig et al., 1996; Jung et al., 2001; Delorme et al., 2012).

Vector autoregressive (VAR) models are versatile tools for analyzing multivariate time series, including multi-channel EEG or multivariate source activities. VAR models predict current values of time series from their recent past (Lütkepohl, 2005). Importantly, they can be used to derive various electrophysiological connectivity measures (Schlögl and Supp, 2006). Volume conduction in biological tissue can be modeled as instantaneous propagation of activity from sources to recording channels. The resulting zero-phase connectivity may be treated as noise added to lagged connectivity patterns of interest. Although some measures, including the imaginary part of the coherency (Nolte et al., 2004), are insensitive to zero-phase connectivity, measures derived from VAR model coefficients do not include such zero-phase terms. Thus, volume conduction effects are not accounted for by the model and affect the correlation structure of the model residuals, which are normally assumed to be uncorrelated.

Popular connectivity measures derived from VAR models include the Directed Transfer Function (DTF) (Kaminski and Blinowska, 1991) and the Partial Directed Coherence (PDC) (Baccalá and Sameshima, 2001). Whereas the PDC is defined in terms of the system matrix (a frequency domain representation of the VAR model), the DTF is based on the inverse of the system matrix. Viewing lagged dependencies between source signals as information flow, the DTF may be said to be normalized by the inflow of information to some sink, while the PDC is normalized by the outflow of information from some source.

As we will demonstrate below, both the DTF and the PDC are indeed adversely affected by volume conduction from multiple sources to the scalp electrodes, in contrast to the claim of Kaminski and Blinowska in their recent opinion article (Kaminski and Blinowska, 2014). Thus, in general direct application of connectivity measures to scalp EEG signals produces less than accurate results and also does not allow their clear interpretation in terms of underlying source dynamics.

## DTF is sensitive to volume conduction

Kaminski and Blinowska (2014) used an example to demonstrate that the DTF is not affected by volume conduction. In their example, they compared the DTF between all pairs of 18 scalp EEG channels to the DTF between the same channels overlaid with a sinusoid at 20 Hz. They interpreted the absence of a peak at 20 Hz in the DTF as evidence for their claim that the DTF is insensitive to volume conduction.

Upon closer inspection, this conclusion turns out to be based on questionable assumptions. First, the figure in their example clearly shows that the DTF obtained from the overlaid sinusoid are not identical to the original DTF. Not only do most of the connectivity measures exhibit troughs at 20 Hz, but the morphologies of some channel pairs are markedly different from the case in which no sinusoid is present. Second, the observed changes in the DTF can be explained by the fact that adding a sinusoid to all EEG channels with equal strength does not reflect volume conduction of a realistic brain source. If anything, their simulation only demonstrates changes in the channel-wise DTF when a single unrelated source signal (here artifactual and non-brain) is present on all channels. Further, their example implicitly confounds scalp and source signals. As described above, volume conduction is more accurately modeled as a mixing process—that is, producing channel activities that are linear combinations of all source activities. Our examples in the following sections will use this model.

## Analytic derivation

We will now demonstrate that non-connected cortical sources generally exhibit spurious connectivity when measured between EEG channels. In general, a VAR model of order *p* can be written as

$$\begin{array}{l} \text{s}\left(t\right)=\overset{p}{\underset{k=1}{\sum }}{\text{A}}_{k}\text{s}\left(t-k\right), \end{array}$$

where **s**(*t*) denotes a multivariate time series, **A**_*k*_ are the model coefficient matrices, and *p* is the model order. For the sake of simplicity, we will use only two sources and a model order of *p* = 1 (but note that the result is valid for an arbitrary number of sources and choice of model order):

$$\begin{array}{l} \text{s}\left(t\right)={\text{A}}_{1}\text{s}\left(t-1\right) \end{array}$$

We begin by setting both off-diagonal elements in the model coefficient matrix to zero, imposing zero causal interaction between the two sources:

$$\begin{array}{l} {\text{A}}_{1}=\left(\begin{array}{cc} a_{11} & 0\\ 0 & a_{22} \end{array}\right) \end{array}$$

Transforming the model to the frequency domain via the Fourier transform yields the system matrix

$$\begin{array}{l} \text{A}\left(\omega \right)=\left(\begin{array}{cc} 1-a_{11}e^{-j\omega } & 0\\ 0 & 1-a_{22}e^{-j\omega } \end{array}\right), \end{array}$$

where ω is the angular frequency and *j*^2^ = −1 is the imaginary unit.

The DTF is based on the transfer matrix **H** of the VAR model, which is the inverse of the system matrix **A**. In our specific example, **H** is therefore also a diagonal matrix:

$$\begin{array}{l} \text{H}\left(\omega \right)={\text{A}}^{-1}\left(\omega \right)=\left(\begin{array}{cc} \frac{1}{1-a_{11}e^{-j\omega }} & 0\\ 0 & \frac{1}{1-a_{22}e^{-j\omega }} \end{array}\right) \end{array}$$

The DTF is just a normalization of the transfer function, which means that it is also a diagonal matrix. It follows that the DTF is zero for both off-diagonal elements, correctly indicating two non-connected sources.

Now we introduce volume conduction via a generic mixing matrix

$$\begin{array}{l} \text{M}=\left(\begin{array}{cc} m_{11} & m_{12}\\ m_{21} & m_{22} \end{array}\right). \end{array}$$

This mixing matrix relates the sources **s**(*t*) to the observed EEG signals **x**(*t*) such that

$$\begin{array}{l} \text{x}\left(t\right)=\text{M}\text{s}\left(t\right). \end{array}$$

Substituting the VAR model into this equation, the mixed model coefficient matrix thus becomes

$$\begin{array}{l} {\tilde{\text{A}}}_{1}=\text{M}{\text{A}}_{1}{\text{M}}^{-1}, \end{array}$$

and the corresponding mixed system matrix

$$\begin{array}{l} \tilde{\text{A}}\left(\omega \right)=\text{I}-{\tilde{\text{A}}}_{1}e^{-j\omega }, \end{array}$$

where **I** is the identity matrix. The mixed transfer function is the inverse of the mixed system matrix

$$\begin{array}{l} \tilde{\text{H}}\left(\omega \right)={\tilde{\text{A}}}^{-1}\left(\omega \right)=\left(\begin{array}{cc} {\tilde{h}}_{11}\left(\omega \right) & {\tilde{h}}_{12}\left(\omega \right)\\ {\tilde{h}}_{21}\left(\omega \right) & {\tilde{h}}_{22}\left(\omega \right) \end{array}\right). \end{array}$$

Plugging in the specific coefficients, the off-diagonal entries of the mixed transfer matrix can be written as

$$\begin{array}{l} {\tilde{h}}_{12}\left(\omega \right)=\frac{m_{11}m_{12}e^{-j\omega }\left(a_{22}-a_{11}\right)}{\left(a_{11}e^{-j\omega }-1\right)\left(a_{22}e^{-j\omega }-1\right)\left(m_{11}m_{22}-m_{12}m_{21}\right)} \end{array}$$

and

$$\begin{array}{l} {\tilde{h}}_{21}\left(\omega \right)=\frac{m_{21}m_{22}e^{-j\omega }\left(a_{11}-a_{22}\right)}{\left(a_{11}e^{-j\omega }-1\right)\left(a_{22}e^{-j\omega }-1\right)\left(m_{11}m_{22}-m_{12}m_{21}\right)}. \end{array}$$

Since the DTF is a specific normalization of the transfer matrix, its off-diagonal elements can only be zero if the corresponding off-diagonal elements of the transfer matrix are zero. In our example, both elements are only zero if *a*_11_ = *a*_22_, that is, if both source activities are exactly equal. This specific case is highly unlikely to occur for real brain sources, which are separated by any distance and concomitant neural conduction delay. In general, the off-diagonal elements will thus be non-zero. This result demonstrates that given non-connected sources, spurious apparent connectivity is introduced into the result of VAR-based connectivity analysis when (as in actual data) volume conduction is present.

## Simulation

Although the analytic derivation can be extended to more than two sources, computing and notating the individual elements of the mixed transfer matrix becomes quite unwieldy. Therefore, we simulated three cortical sources and used a realistic forward model of volume conduction from brain to scalp to numerically estimate their summed activity at three scalp EEG channels. For this simulation, we used the freely available Brainstorm toolbox (Tadel et al., 2011); our analyses are based on the ICBM152 template brain (Mazziotta et al., 1995) included in the toolbox. The source code for this analysis can be found in the Supplementary Material section. Once again, we start by specifying the ground truth, here the connectivity structure of the cortical sources consisting of one active connection from Source 1 to Source 3. The corresponding model coefficient matrix is

$$\begin{array}{l} {\text{A}}_{1}=\left(\begin{array}{ccc} 0.9 & 0 & 0\\ 0 & 0.5 & 0\\ 1 & 0 & -0.5 \end{array}\right). \end{array}$$

**Figure 1:**
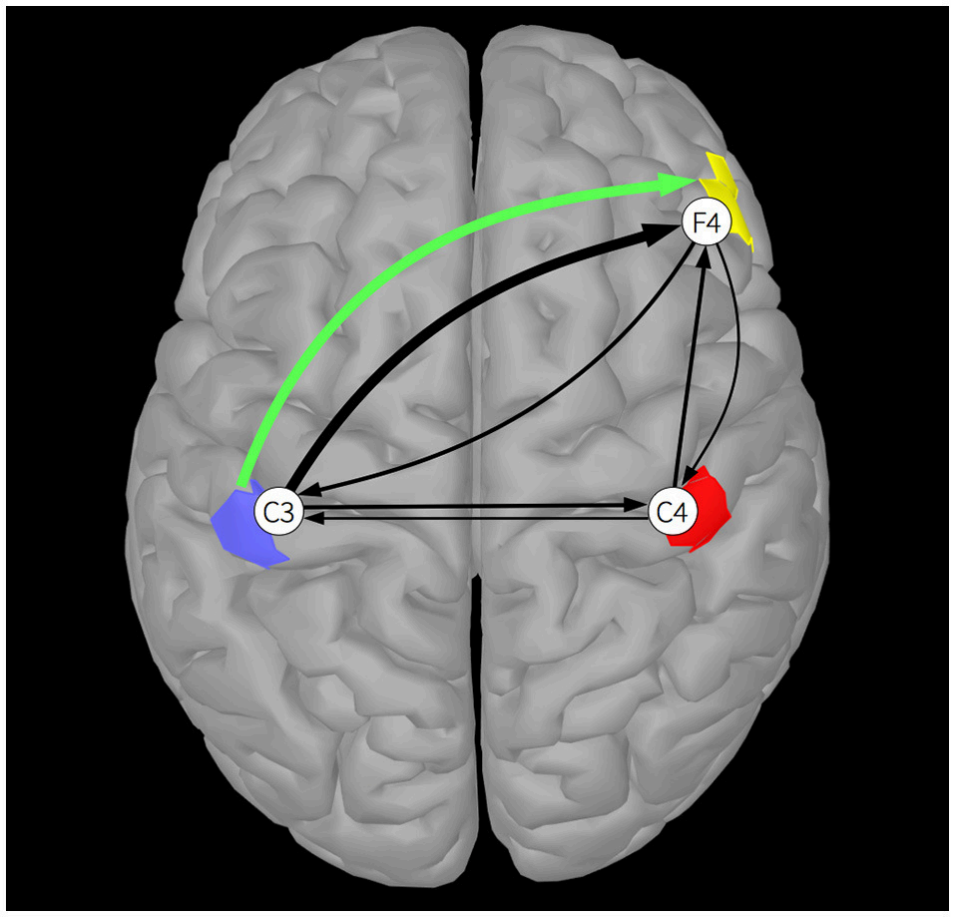
**A single DTF connection, averaged across all frequencies, between two of the three cortical sources (blue, red, and yellow patches), is shown by a green arrow**. Black arrows indicate DTF connections estimated between EEG electrode channels C3, C4, and F4. The widths of the arrows are proportional to their DTF strength.

Next, we assume these three sources exhibit spatially coherent activity across upward-facing (gyral) cortical patches located just below electrodes placed at C3, C4, and F4 (see Figure 1). We compute the forward solution from the realistic template head model to obtain the mixing matrix **M** containing the weights that mix the activities of the three sources to the three scalp channels:

$$\begin{array}{l} \text{M}=(\begin{array}{ccc} 25.62 & 1.51 & -3.77\\ 1.25 & 19.70 & 2.68\\ 0.64 & 7.84 & 30.95 \end{array}) \end{array}$$

The matrix of mixed coefficients is

$$\begin{array}{l} {\tilde{\text{A}}}_{1}=\text{M}{\text{A}}_{1}{\text{M}}^{-1}=\left(\begin{array}{ccc} 0.75 & -0.08 & 0.16\\ 0.12 & 0.52 & -0.07\\ 1.23 & 0.25 & -0.37 \end{array}\right). \end{array}$$

The corresponding source DTF averaged over all frequencies is given by

$$\begin{array}{l} {\bar{\text{DTF}}}_{\text{source}}=\left(\begin{array}{ccc} 1 & 0 & 0\\ 0 & 1 & 0\\ 0.66 & 0 & 0.70 \end{array}\right), \end{array}$$

whereas the corresponding channel DTF is given by

$$\begin{array}{l} {\bar{\text{DTF}}}_{\text{channels}}=\left(\begin{array}{ccc} 0.98 & 0.08 & 0.16\\ 0.14 & 0.98 & 0.08\\ 0.72 & 0.17 & 0.62 \end{array}\right). \end{array}$$

Figure 1 shows the results of the simulation. The green arrow indicates the ground truth connection between Source 1 (blue patch) and Source 3 (yellow patch). The black arrows visualize the estimated EEG channel connectivities. The width of the arrows is proportional to the strength of the DTF across all frequencies. Once again, it is apparent that volume conduction and summation of the projected signals at the scalp electrodes produces spurious connectivity results. Whereas there is only one ground truth source-to-source connection, non-zero values are produced between all channel pairs by applying DTF to the channel data. Note that spurious channel-to-channel connections are caused by volume conduction and not by indirect connections—similar connectivity patterns can also be observed in PDC estimates of channel-to-channel connections. Here, the dominant estimated channel-to-channel connectivity is between simulated electrode channels placed directly above the coupled sources. In simulations in which the unknown source locations are more distant from the scalp electrodes, channel connectivity estimates may not suggest a particular source connectivity.

## Discussion

We have demonstrated both analytically and by numerical simulation that the DTF is influenced by volume conduction. In contrast to Kaminski and Blinowska (2014), we argue that connectivity measures should generally be calculated on cortical sources, because they are the actual sources of brain activity. Ideally, source activities can be obtained by separating each data channel signal into a sum of physically and physiologically distinct source processes whose interrelationships can also be modeled in terms of causal connectivity.

It is worth mentioning that we selected highly favorable EEG channel and source locations in our simulation example. Even in this best-case scenario, spurious connections arise between all channel pairs. However, most cortical sources do not necessarily project radially from cortical gyri to scalp locations just above them—with different source locations, we could have obtained a collection of suggested scalp channel connections far from the originating sources, with no way to determine which source connections contributed to what extent.

In conclusion, while DTF and other connectivity estimators can be applied to either scalp channels or to (estimated) source signals, results are highly likely to be more accurate when the analysis is based on source activities. In addition, interpreting the results correctly in terms of regional brain interactions is also much more straightforward. This is because connectivity computed between EEG channels is heavily confounded by the broad volume conduction patterns associated with each regional EEG source.

## Author contributions

CB led the article (which includes writing and contributing to all aspects of the article such as the analytic proof and the simulation example), MB laid out the analytic proof, MS contributed the simulation, TM provided input and comments/corrections on all aspects of the article, and SM provided input and comments as well as rewrote several paragraphs of the article.

### Conflict of interest statement

The authors declare that the research was conducted in the absence of any commercial or financial relationships that could be construed as a potential conflict of interest.
